# Cone Beam CT-Based Preoperative Volumetric Estimation of Bone Graft Required for Lateral Window Sinus Augmentation, Compared with Intraoperative Findings: A Pilot Study

**DOI:** 10.2174/1874210601812010820

**Published:** 2018-10-25

**Authors:** Nagla'a A. Abdel-Wahed, Maha Ahmed Bahammam

**Affiliations:** 1 Department of Oral Diagnostic Sciences, Faculty of Dentistry, King Abdulaziz University, Jeddah, Saudi Arabia; 2 Department of Periodontology, Faculty of Dentistry, King Abdulaziz University, Jeddah, Saudi Arabia

**Keywords:** Volumetric estimation, Bone graft, Sinus augmentation, Dental implants, Cone beam CT, Pilot study

## Abstract

**Introduction:**

The presence of an atrophic maxilla creates a serious challenge in cases of implant placement, while maxillary sinus pneumatization further complicates the surgery. This pilot study was performed to investigate the validity of two techniques used to estimate the volumes of bone graft material required in cases that included lateral window sinus augmentation.

**Materials and Methods:**

Cone beam computed tomography was used for preoperative volumetric analysis of the maxillary sinus. The analysis was performed using the manual measurement of sinus dimensions, as well as automated measurements* via *the segmentation technique. The estimated volumes of required bone graft material were compared with actual intraoperative findings in cases requiring lateral window sinus augmentation. For this pilot study, only 5 patients were selected to be included.

**Results:**

To achieve 80% power and confidence interval of 95%, the sample size should be 35 patients. The correlation coefficient between the segmented volume and mm^3^ used was – 0.5332, whereas the coefficient between the manual volume and mm^3^ used was – 0.6784. Consequently, both results indicate that the two methods have a moderate negative correlation with the mm^3^ used.

**Conclusion:**

Performing a similar study with an increased number of patients, according to the calculated sample size, increases the possibility of revealing higher correlation between the methods used to analyze the partial volume of the sinus cavity. The estimated sinus volume of the area of augmentation, obtained by using either manual or segmentation techniques, could be considered as a maximum estimate for the required amount of graft material. Furthermore, the segmentation technique may be valuable in preoperative planning of sinus augmentation, as it reveals the topographic shape and morphology of the sinus.

## 1 INTRODUCTION

The presence of an atrophic maxilla creates a serious challenge in cases of implant placement, whereas maxillary sinus pneumatization further complicates the surgery. Patients who exhibit both conditions require bone grafting to the maxillary sinus floor to increase the height of the maxillary alveolar bone [1-7]. Lateral window sinus lift or Schneiderian membrane elevation is a common surgical procedure, first published by Boyne and James in 1980, that is used to create a window on the lateral wall of the sinus, as well as a space between the Schneiderian membrane and the sinus walls for the placement of grafting materials. Lateral window sinus lift is an effective procedure to gain bone height to facilitate future implant placement in an atrophic pneumatized posterior maxilla [1].

Preoperative planning includes radiographic assessment of maxillary sinus dimensions. Digital panoramic radiographs have been used [8], but they exhibit no ability to investigate depth. Conventional dental or panoramic radiographs are not suitable for volumetric analysis, as their two-dimensional nature only provides an approximation of sinus graft vertical dimensions. Thus, 3D imaging is required to allow investigation of the presence of bony septa, as well as Schneiderian membrane thickness and residual alveolar bone height [9, 10], thereby allowing the proper design of the lateral wall sinus augmentation.

Several previous studies have used Computed Tomography (CT) as a method for sinus anatomy evaluation and volume calculation [11-14]. Cone Beam CT (CBCT) has also been used with comparable accuracy [9, 15-18].

Generally, the need for decreased postoperative complications at the donor site (during autogenous graft surgeries) necessitates careful preoperative planning of the graft volume to be harvested. The increased cost of allografts leads to further urgency in preoperative planning.

This pilot study was performed to investigate the validity of two techniques used to estimate volumes of bone graft material, required in cases that included lateral window sinus augmentation, through CBCT imaging. The null hypothesis is that there is no statistical significance between the estimated volumes and actual intraoperative findings.

## 2 MATERIALS AND METHODS

This pilot study included patients who presented to the Department of Periodontology at King Abdulaziz University for the placement of dental implants in the maxillary posterior area. Patients were interviewed and examined to determine their eligibility for the study. Inclusion criteria were as follows: consent for the described procedure as approved by the University’s Institutional Ethical Committee on Human Research, the requirement for lateral wall sinus augmentation prior to dental implant placement, and age ≥18 years. Exclusion criteria were as follows: Ongoing pregnancy, diagnosis with metabolic disorder, immunocompromised status, hemophilia, bleeding disorders, drug or alcohol abuse, treatment with steroids, history of radiation therapy in the head and neck, psychiatric disorders, and/or inability to understand the procedure described in the questionnaire. The statistical tool determined the sample size, which was 35 patients at 80% power and confidence interval of 95%. For this pilot study, only 5 patients were selected to be included.

3D imaging was performed before lateral wall sinus augmentation. CBCT images were acquired using a Next Generation i-CAT scanner (Imaging Sciences International, Inc., Hatfield, USA). A scout (preview) was obtained and adjustments were made to ensure that the patient was correctly aligned in the scanner before acquisition, using the adjustment light beam. The machine was supplied with an Amorphous Silicon Flat Panel Sensor with Cesium Iodide (CsI) scintillator (0.5-mm focal spot size, 14-Bit grayscale resolution) and operated at the following protocol for all the scans of the study: 120 kVp, 37.07 mAs, 8.9 s, 0.4 mm Voxel size, and 13 × 16 cm^2^ FOV.

Following acquisition, data were exported and transferred in DICOM format, then downloaded* via *a Compact Disk (CD) to a personal computer for volume measurement, where Mimics software (version 15.1; Materialize, Belgium) was utilized.

Data were imported into Mimics software; at the coronal view, the edentulous site was viewed, and a 12-mm vertical linear measurement was made from the crest of the ridge upwards (at the area of minimum dimension). Next, the axial reference line was moved to the site at the end of the previous measurement; this level was assigned as the level up to which segmentation of the maxillary sinus space should reach. A certain threshold was assigned to segment the sinus space; then cropping and region-growing tools were used to confine the segmented mask to the maxillary sinus space. Final fine-tuning and clean-up steps were performed at the axial cut sites to ensure the geometry of the segmented mask. After calculating the volume of the segmented mask, the software presented the amount of graft material required to augment the sinus, thereby reaching 12-mm bone height [15].

Additionally, manual measurements were performed of the sinus dimensions and volume calculation. These were dependent on the anatomical fact that the maxillary sinus is pyramidal in shape, with an almost square base that is oriented medially [16]. At the coronal view, the edentulous site was viewed, and a 12-mm vertical linear measurement was taken from the crest of the ridge upwards (at the area of minimum dimension). Next, the axial reference line was moved to the site at the end of the previous measurement; this level was assigned as the level up to which linear measurements will be performed. To obtain the width, length, and height of the sinus, the coronal, axial, and sagittal cuts were sequentially reviewed to acquire maximum sinus mediolateral, anteroposterior, and craniocaudal dimensions.

Finally, radiographic data was compared to intraoperative findings and statistically evaluated by correlation analysis.

This study was approved by the Research Ethics Committee of the Faculty of Dentistry, King Abdulaziz University (No. 009-16).

## 3 RESULTS

The statistical tool determined the sample size, which was 35 patients at 80% power and confidence interval of 95%.

Results obtained from this pilot study done for 5 patients are as follows: Table **1** represents the correlation coefficient between each of the two methods and mm^3^ used. The correlation coefficient between the mimics volume and mm^3^ used was – 0.5332, whereas the coefficient between the manual volume and mm^3^ used was – 0.6784. The resulting linear regression models were as follows:

Estimated mm^3^ used = – 0.1865 × Mimics volume + 2094.5

Estimated mm^3^ used = – 0.4162 × manual volume + 2149

The two models above were used to estimate the mm^3^ used, based on the given Mimics volume and manual volume values. The residual error was measured and normalized to obtain the coefficient of determination (*i.e*., R^2^) of the model. The calculated R^2^ values for the two models above were 0.2843 and 0.4603, respectively.

Figs. (**1** and **2**) depict snapshots of manual and Mimics volumetric calculations for the patients.

## 4 DISCUSSION

In this pilot study, CBCT was used for preoperative volumetric analysis of the maxillary sinus. The estimated volumes of required bone graft material were compared with actual intraoperative findings. Although the determined sample size was 35, considering the nature of this study (pilot), number of patients was limited to five.

To assess the ability of the measured volume parameters to accurately predict the actual mm^3^ used, the correlation coefficient was evaluated between each of the two methods and the mm^3^ used. Briefly, the correlation generally measures the existence and strength of a linear model between two variables; the correlation coefficient used produces results between +1 (strong positive linear dependence) and -1 (strong negative linear dependence), with 0 indicating no relationship between the two variables. Thus, the closer the magnitude of the correlation coefficient to 1, the more plausible a linear model is to describe the relationship. The results indicate that the two methods have a moderate negative correlation with the mm^3^ used.

The next step in estimating the validity of the linear model was to perform a linear regression to generate an estimate of the mm^3^ used, from the value of either the Mimics volume or the manual volume. Furthermore, to check the goodness-of-fit of the resulting linear models, both models were used to estimate the mm^3^ used, based on the given Mimics volume and manual volume values. The residual error was measured and normalized to obtain the coefficient of determination (*i.e*., R^2^) of the model. This metric has a value between 0 and 1, where 1 designates a perfect linear model. Hence, the larger the value of R^2^, the better the model to accurately predict the data.

The results of this pilot study were more towards rejecting the null hypothesis. Performing a similar study according to the calculated sample size, may reveal a higher correlation between the methods used to analyze the partial volume of the sinus cavity and actual intraoperative findings. However, in this study, the model based on manual volume exhibited a much better goodness-of-fit.

In a previous study [16], manual calculation of the whole sinus used the laws of geometry to give an estimated volume, which significantly correlated with the estimation* via *segmentation technique. Previous studies also incorporated manual or automated segmentation to assess sinus or grafted bone volume [13, 14, 19, 20]. Uchida * et al*. [13, 14] investigated the volume of grafted bone required for sinus floor lifting and determined a range of 4.74-7.96 cm^3^ graft volume, depending on the height of required sinus floor lift, which ranged from 15-20 mm. It has been also reported that the calculated augmentation volume for an augmentation height of 12 mm was 1.7 ± 0.9 cm^3^ [21]. These ranges reasonably consistent with the results of this study, as the level of lift was set at 12 mm.

The segmentation technique by Mimics provides additional advantages, as it reveals the topographic shape and morphology of the sinus, thereby informing the surgeon of the anatomical limitations expected. However, the inaccuracy of the model’s goodness-of-fit cannot be disregarded. Thus, the estimated volumes should be used only as a guide. The greater value of the Mimics method (or any other segmentation technique) lies in its accurate morphological representation of sinus anatomy. The resulting 3D shapes provide an ideal preoperative guide for the clinician.

## CONCLUSION

Performing a similar study with an increased number of patients, according to the calculated sample size, increases the possibility of revealing higher correlation between the methods used to analyze partial volume of the sinus cavity. The estimated sinus volume of the area of augmentation, obtained by using either manual or segmentation techniques, could be considered as a maximum estimate for the required amount of graft material. Furthermore, the segmentation technique may be valuable in preoperative planning of sinus augmentation, as it reveals the topographic shape and morphology of the sinus.

## Figures and Tables

**Fig. (1):**
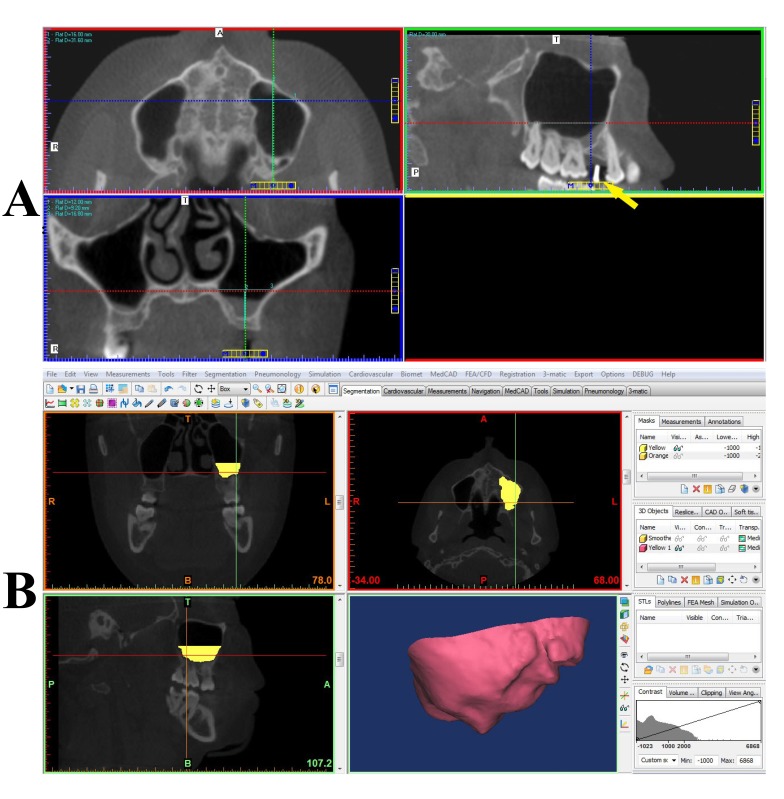
**A:** Snapshot showing the linear measurements used to calculate the sinus volume for a patient (yellow arrow points at the radiopaque marker inserted in the imaging stent); **B:** Snapshot showing calculation of the sinus volume for the same patient, performed with the Mimics program.

**Fig. (2):**
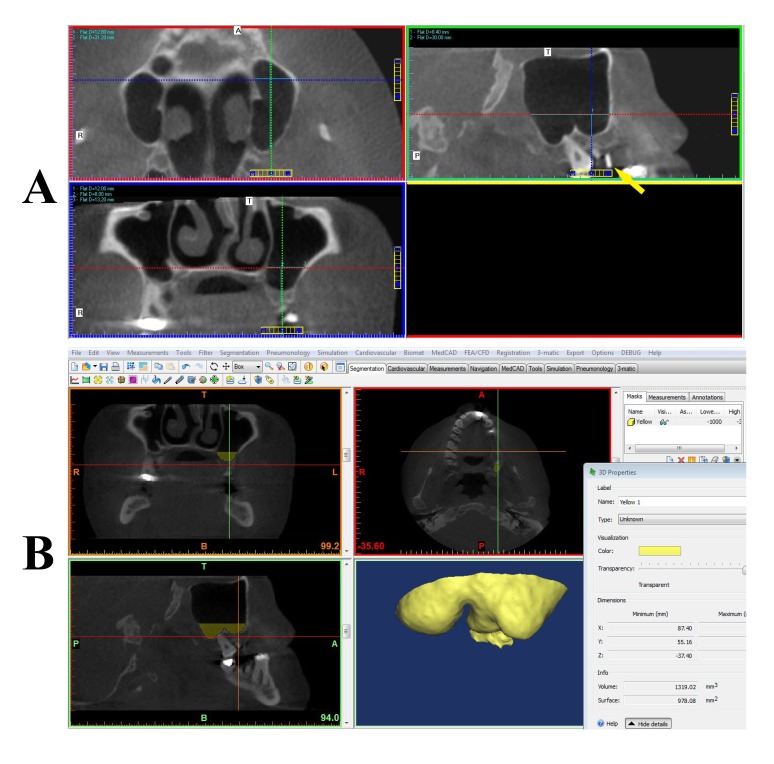
**A:** Snapshot showing the linear measurements used to calculate the sinus volume for a patient (yellow arrow points at the radiopaque marker inserted in the imaging stent); **B:** Snapshot showing calculation of the sinus volume for the same patient, performed with the Mimics program.

**Table 1:** Maxillary sinus volumetric analysis.

**Actual Volume of Material Used** **(mm^3^)**	**Estimated Volume from Mimics** **(mm^3^)**	**Estimated Volume from Manual Calculation** **(mm^3^)**
1500	2179	1030.36
2000	1997	396.03
2000	1319	1098.24
1500	1693	1241.86
1500	3389	1628.03
***r***	**-0.5332**	**-0.6784**

## LIST OF ABBREVIATIONS

CT: = Computed Tomography
CBCT: = Cone Beam Computed Tomography
CsI: = Cesium Iodide
CD: = Compact Disk

## References

[1] Boyne P.J., James R.A. (1980). Grafting of the maxillary sinus floor with autogenous marrow and bone.. J. Oral Surg..

[2] Hochwald D.A., Davis W.H., Worthington P., Brånemark P.I. (1992). Bone grafting in the maxillary sinus floor.. Advanced Osseointegration Surgery: Application in the Maxillary Region..

[3] Isaksson S. (1994). Evaluation of three bone grafting techniques for severely resorbed maxillae in conjunction with immediate endosseous implants.. Int. J. Oral Maxillofac. Implants.

[4] Keller E.E., Eckert S.E., Tolman D.E. (1994). Maxillary antral and nasal one-stage inlay composite bone graft: Preliminary report on 30 recipient sites.. J. Oral Maxillofac. Surg..

[5] Kent J.N., Block M.S. (1989). Simultaneous maxillary sinus floor bone grafting and placement of hydroxylapatite-coated implants.. J. Oral Maxillofac. Surg..

[6] Li K.K., Stephens W.L., Gliklich R. (1996). Reconstruction of the severely atrophic edentulous maxilla using Le Fort I osteotomy with simultaneous bone graft and implant placement.. J. Oral Maxillofac. Surg..

[7] Sailer H.F. (1989). A new method of inserting endosseous implants in totally atrophic maxillae.. J. Craniomaxillofac. Surg..

[8] Machtei E.E., Rozitky D., Zigdon-Giladi H., Horwitz J. (2016). Dimensional changes following lateral wall sinus augmentation without concomitant implant placement using a composite bone graft.. Int. J. Oral Maxillofac. Implants.

[9] Gurler G., Delilbasi C. (2015). Relationship between preoperative cone beam computed tomography and intraoperative findings in sinus augmentation.. Int. J. Oral Maxillofac. Implants.

[10] Tadinada A., Jalali E., Al-Salman W., Jambhekar S., Katechia B., Almas K. (2016). Prevalence of bony septa, antral pathology, and dimensions of the maxillary sinus from a sinus augmentation perspective: A retrospective cone-beam computed tomography study.. Imaging Sci. Dent..

[11] Ariji Y., Ariji E., Yoshiura K., Kanda S. (1996). Computed tomographic indices for maxillary sinus size in comparison with the sinus volume.. Dentomaxillofac. Radiol..

[12] Ariji Y., Kuroki T., Moriguchi S., Ariji E., Kanda S. (1994). Age changes in the volume of the human maxillary sinus: A study using computed tomography.. Dentomaxillofac. Radiol..

[13] Uchida Y., Goto M., Katsuki T., Akiyoshi T. (1998). A cadaveric study of maxillary sinus size as an aid in bone grafting of the maxillary sinus floor.. J. Oral Maxillofac. Surg..

[14] Uchida Y., Goto M., Katsuki T., Soejima Y. (1998). Measurement of maxillary sinus volume using computerized tomographic images.. Int. J. Oral Maxillofac. Implants.

[15] Hamdy R.M., Abdel-Wahed N. (2012). Cone-beam computed tomographic volumetric analysis of the maxillary antra for sinus augmentation.. Egypt. Dent. J..

[16] Hamdy R.M., Abdel-Wahed N. (2014). Three-dimensional linear and volumetric analysis of maxillary sinus pneumatization.. J. Adv. Res..

[17] Loubele M., Van Assche N., Carpentier K., Maes F., Jacobs R., van Steenberghe D., Suetens P. (2008). Comparative localized linear accuracy of small-field cone-beam CT and multislice CT for alveolar bone measurements.. Oral Surg. Oral Med. Oral Pathol. Oral Radiol. Endod..

[18] Neugebauer J., Ritter L., Mischkowski R.A., Dreiseidler T., Scherer P., Ketterle M., Rothamel D., Zöller J.E. (2010). Evaluation of maxillary sinus anatomy by cone-beam CT prior to sinus floor elevation.. Int. J. Oral Maxillofac. Implants.

[19] Gultekin BA, Borahan O, Sirali A, Karabuda ZC, Mijiritsky E (2016). Three-dimensional assessment of volumetric changes in sinuses augmented with two different bone substitutes.. Biomed Res Int.

[20] Okada T., Kanai T., Tachikawa N., Munakata M., Kasugai S. (2016). Long-term radiographic assessment of maxillary sinus floor augmentation using beta-tricalcium phosphate: Analysis by cone-beam computed tomography.. Int. J. Implant Dent..

[21] Krennmair G., Krainhöfner M., Maier H., Weinländer M., Piehslinger E. (2006). Computerized tomography-assisted calculation of sinus augmentation volume.. Int. J. Oral Maxillofac. Implants.

